# Tailoring evidence to clinical practice

**DOI:** 10.1007/s12471-025-01928-5

**Published:** 2025-01-20

**Authors:** Pim van der Harst

**Affiliations:** Department of Cardiology, Division of Heart and Lungs, University Medical Centre Utrecht, Utrecht, The Netherlands

This February issue of the *Netherlands Heart Journal* features three important articles that highlight the intersection between evidence-based guidelines, novel diagnostic approaches, and patient-centered care in cardiology.

Maass et al. report on the Netherlands Society of Cardiology (NVVC) endorsement of the 2021 ESC guidelines on cardiac pacing and cardiac resynchronisation therapy [1]. This work highlights the integration of shared decision-making, reflecting the priorities of Dutch clinical practice, especially in settings with limited clinical evidence. Key adaptations include aligning recommendations with Dutch guidelines, such as the selective use of CRT without defibrillator functionality for non-ischemic cardiomyopathy, and the incorporation of MRI safety protocols for patients with implantable cardiac devices. Emerging techniques, such as conduction system pacing and leadless pacemakers, are also discussed as promising solutions to current challenges, pending further randomised trial data.

Thierry et al. provide data on a decade of family screening for dilated cardiomyopathy (DCM) [2], demonstrating the benefits of a genetics-first approach. Their findings confirm that relatives without familial (likely) pathogenic variants can safely omit further cardiac screening, thereby optimising resource use. The study underscores the importance of targeted screening in preventing adverse outcomes and align with the growing emphasis on personalised care.

Van der Velde et al. evaluated advanced imaging techniques in cardiac sarcoidosis [3], demonstrating the diagnostic superiority of cardiovascular magnetic resonance and FDG-PET compared to traditional methods. However, patients with cardiac sarcoidosis who have normal ECG and TTE findings have a favourable prognosis, highlighting the importance of further studies to personalised diagnostic and follow-up strategies.

Together, these three contributions underscore the importance of tailoring evidence-based guidelines and novel diagnostic strategies to patient preferences to enhance cardiac care in the Netherlands.
